# Effects of Hops Treatment on Nitrogen Retention, Volatile Fatty Acid Accumulations, and Select Microbial Populations of Composting Poultry Litter Intended for Use as a Ruminant Feedstuff

**DOI:** 10.3390/microorganisms11040839

**Published:** 2023-03-25

**Authors:** Yamicela Castillo-Castillo, Claudio Arzola-Alvarez, Mozart Fonseca, Jaime Salinas-Chavira, Marina Ontiveros-Magadan, Michael E. Hume, Robin C. Anderson, Michael D. Flythe, James Allen Byrd, Oscar Ruiz-Barrera

**Affiliations:** 1 Facultad de Zootecnia y Ecología, Universidad Autónoma de Chihuahua, Chihuahua 31453, Mexico; 2 Department of Agriculture, Veterinary & Rangleland Sciences, University of Nevada, Reno, NV 89154, USA; 3 Facultad de Medicina Veterinaria y Zootecnia—Nutricion Animal, Universidad Autónoma de Tamaulipas, Ciudad Victoria 87000, Mexico; 4 Departamento de Medicina Veterinaria y Zootecnia, Universidad Autónoma de Ciudad Juárez, Juarez 32310, Mexico; 5 Food and Feed Safety Research Unit, Agricultural Research Service, United States Department of Agriculture, College Station, TX 77845, USA; 6 Forage-Animal Production Research Unit, Agricultural Research Service, United States Department of Agriculture, Lexington, KY 40506, USA

**Keywords:** composting, crude protein, hops, microbial pathogens, poultry litter

## Abstract

Poultry litter is a valuable crude protein feedstuff for ruminants, but it must be treated to kill pathogens before feeding. Composting effectively kills pathogens, but it risks losing ammonia to volatilization or leaching during degradation of uric acid and urea. Hops bitter acids also exert antimicrobial activity against certain pathogenic and nitrogen-degrading microbes. Consequently, the present studies were conducted to test if adding bitter acid-rich hop preparations to simulated poultry litter composts may improve nitrogen retention while simultaneously improving pathogen killing. Results from an initial study, testing doses of Chinook or Galena hops preparations designed to each deliver 79 ppm hops β-acid, revealed that, after nine days simulated composting of wood chip litter, ammonia concentrations were 14% lower (*p* < 0.05) in Chinook-treated composts than untreated composts (13.4 ± 1.06 µmol/g). Conversely, urea concentrations were 55% lower (*p* < 0.05) in Galena-treated than untreated composts (6.2 ± 1.72 µmol/g). Uric acid accumulations were unaffected by hops treatments in this study but were higher (*p* < 0.05) after three days than after zero, six, or nine days of composting. In follow-up studies, Chinook or Galena hops treatments (delivering 2042 or 6126 ppm of β-acid, respectively) for simulated composts (14 days) of wood chip litter alone or mixed 3:1 with ground Bluestem hay (*Andropogon gerardii*) revealed that these higher dosages had little effect on ammonia, urea, or uric acid accumulations when compared to untreated composts. Volatile fatty acid accumulations measured in these later studies were affected by the hops treatments, with butyrate accumulations being lower after 14 days in hops-treated composts than in untreated compost. In all studies, beneficial effects of Galena or Chinook hops treatments were not observed on the antimicrobial activity of the simulated composts, with composting by itself decreasing (*p* < 0.05) counts of select microbial populations by more than 2.5 log_10_ colony forming units/g compost dry matter. Thus, while hops treatments had little effect on pathogen control or nitrogen retention within the composted litter, they did lessen accumulations of butyrate, which may prevent adverse effects of this fatty acid on palatability of litter fed to ruminants.

## 1. Introduction

Disposal of used poultry litter can be environmentally challenging for producers, as repeated application to land as a fertilizer can cause excessive accumulations of phosphorous, potassium, and nitrogen in the soil or watershed [1,2]. Poultry litter contains appreciable amounts of its nitrogen as uric acid and urea, which makes it an excellent crude protein supplement for feedstuffs fed to ruminants whose rumen microbial population can transform and thus upgrade these nitrogen sources into microbial protein of nutritional value to the host [3,4]. Poultry litter can contain high numbers of bacterial pathogens, such as *Salmonella enterica*, *Escherichia coli*, and *Campylobacter jejuni*, as well as antimicrobial resistant microbes and thus must be treated before being fed to ruminants [5,6]. Biotechnological processes, such as composting and ensiling, are cost-effective technologies that can kill pathogens in poultry litter; however, the process can result in the breakdown of uric acid, urea, and other fixed nitrogen sources related to ammonia, which, depending on pH, can be lost during storage due to volatilization to the atmosphere or leaching to the surrounding ground environment [7,8,9,10,11]. Consequently, there is interest in developing technology capable of preserving the crude protein content of poultry litter during composting.

The addition of certain natural products, such as biochar, lignite, tannins, or certain secondary plant compounds, have been studied as potential supplements to composting litter to prevent ammonification of the crude protein. For instance, addition of pine chip biochar to composting poultry litter was reported to improve nitrogen retention, purportedly via binding of ammonia precursors, such as uric acid, urea, and ammonium ions [12,13]. The binding of ammonia by supplemental lignite was also reported to indirectly inhibit urease-catalyzed conversion of ammonium ions to ammonia and to inhibit propagation of ammonia-oxidizing microbial populations via limitation of substrate availability [13]. Similarly, the addition of pine bark tannin extracts to composting poultry litter increased uric acid accumulation by 62% and decreased ammonia accumulation by 23% compared to untreated controls after nine days of composting [14]. Mechanistically, tannins are recognized for their protein-binding activity and their ability to inhibit important amino acid-degrading microbes [15]. Tannins are also reported to inhibit xanthine oxidase activity, which catalyzes the reversible transformation of xanthine to uric acid [16]. The addition of a phytochemical, 3-nitropropionate, produced by certain forages and fungi, as well as certain other xenobiotic nitrocompounds, has resulted in increased uric acid retention and decreased ammonia accumulations after nine days of composting [17]. These nitrocompounds are thought to interrupt anaerobic degradation of uric acid by inhibiting ferredoxin-mediated electron transfer reactions involved in anaerobic degradation of uric acid [17,18]. Overall, the decrease in ammonia accumulations likely reflects an increased retention of ammonia incorporated into uric acid. However, increased retention of ammonia in amino acids due to decreased microbial degradation or increased microbial synthesis of amino acids cannot be ruled out. However, the effects of the tannin- or nitro-treatments on urea accumulations have been inconsistent, possibly due to the role of urea as an intermediate between uric acid degradation and ammonia production [17,19], which currently limits their application in practice.

Pine bark tannin extracts or some short chain nitrocompounds supplemented to composted poultry litter also contributed modestly to the anti-*Salmonella* activity of the composting process. From a practical perspective, however, the slight enhancement in antimicrobial activity of the tannin or nitrocompound treatments is likely of limited benefit, as the composting process by itself usually eliminates *Salmonella* after 14 days of composting [6,20].

Hops (*Humulus lupulus*), long used by the brewing industry and in traditional medicine, contain abundant amounts of prenylated phloroglucinol compounds, referred to as hops bitter acids [21]. These bitter acids, comprised of α-acids or β-acids (lupulone, co-lupulone, and ad-lupulone), are reported to exhibit antimicrobial activity against a variety of microbes and particularly against Gram-positive pathogens often found in gut habitats or excreta. These pathogens include members of *Clostridium*, *Listeria*, *Streptococcus*, *Staphylococcus*, as well as important hyper ammonia-producing bacteria, such as *Clostridium aminophilum* and *Acetoanaerobium sticklandii*, as well as *Peptoniphilus indolicus* [21,22,23,24,25,26,27]. While the hyper ammonia-producing bacteria are recognized as playing a major role in decreasing nitrogen retention in ruminants [28], their contribution to nitrogen metabolism within poultry or poultry litter is unclear. Considering, however, that many of the bacterial species inhibited by the bitter acids are important ureolytic organisms and that hop components are reported to inhibit urease activity [29,30], it seems reasonable to hypothesize that supplementing composting poultry litter with hops may aid the retention of crude protein in the composted feedstuff.

Accordingly, the primary objective of the present study was to assess the effects of Chinook or Galena hops on uric acid, urea, and ammonia accumulations during composting while subordinately assessing their effects on select microbial populations and volatile fatty acid accumulations. 

## 2. Materials and Methods

### 2.1. Poultry Litter, Ground Forage and Hop Extracts

Used wood chip poultry litter used in the first experiment was provided by the Texas A&M University Poultry Science Department (College Station, TX, USA); this litter was approximately one-year-old and had been used to rear seven flocks of broilers. Wood chip poultry litter used in the second and third experiments was collected at the end of a 48-day grow-out period from a broiler flock reared at the Southern Plains Agricultural Research Center poultry facility in College Station, TX, USA. Neither batch of litter had any known exposure to antibiotics or coccidiostats. Prior to use in simulated compost experiments, each litter batch was screened through a 17-mm diameter sieve to omit oversized particles. The bluestem hay used in the third experiment had been ground to pass a 100-mm screen and was provided by the Texas A&M Animal Science Department. The dry matter (DM) content of the first and second batch of screened litter, as determined by drying three replicate subsamples at 100 °C for 24 h, was 76.3 ± 0.2% and 88.4 ± 4.3%, respectively. The dry matter of the ground bluestem hay, determined likewise, was 92.2 ± 0.3%. The composition of the bluestem hay, as determined by the Texas A&M AgriLife Extension Service Soil, Water and Forage Testing Laboratory (soiltesting.tamu.edu), was 4.8% crude protein, 39.8% acid detergent fiber, 52.5% total digestible nutrients-based acid detergent fiber, and 0.53, 0.56, and 0.23 Mcal/kg net energy for lactation, maintenance, and gain, respectively. The Chinook hops and Galena hops pellets used for both compost simulations were provided by Lupulin Exchange (Charlottesville, VA, USA) and their composition, with α-acid contents of about 12 and 13%, and β-acid contents of about 3 and 9%, respectively, have been described earlier [31]. The α-acids and β-acids from hops exhibit antimicrobial properties [23,31,32], with β-acids more specifically affecting amino acid-degrading, hyper ammonia-producing bacteria [33].

### 2.2. Design of Compost Simulations

In Experiment 1, 250-g portions of screened poultry litter were spread to cover the bottoms of four separate 26 × 39 × 8 cm plastic trays. Each tray was then thoroughly mixed with 125 mL of 0.4 M phosphate buffer (pH 6.5) alone or phosphate buffer suspensions containing 0.50 g of Chinook hops or 0.167 g of Galena hops to achieve on a dry matter basis an effective coverage of 0.26% (2618 ppm) or 0.09% (874 ppm) of the respective hops, which equates to approximately 0.03 and 0.01% (314 and 114 ppm, respectively) of α-acid or 0.008% (79 ppm) β-acid/g compost dry matter, respectively. These doses were made equivalent in β-acid content to be slightly higher than the 60 ppm dose used by Flythe and Aiken [34]. The poultry litter mixtures were then each hand-mixed, and then 11-g portions were transferred to 50-mL plastic tubes (12 tubes/treatment to achieve four tubes/treatment per sample day) and amended with 1.1 mL of phosphate buffer containing 10^3^ colony forming units (CFU)/mL of an overnight challenge culture (at 37 °C in tryptic soy broth; Difco, Becton Dickinson, Sparks, MD, USA) of *Salmonella enterica* serovar Typhimurium previously made novobiocin- and nalidixic acid-resistant [35]. The tubes were closed with caps, sealed with parafilm, and incubated at successive three-day temperature increments at 22 °C, 37 °C, and 42 °C, respectively, to simulate conditions commonly encountered during composting. For all incubations, the tubes were placed in BBL Anaerobic Gas Pack (Becton Dickinson) containers and incubated aerobically during days 0 to 3 then flushed with 100% with carbon dioxide and incubated under this atmosphere for days 4 to 9.

In Experiment 2, 11-g portions of the second batch of screened poultry litter were mixed without or with 0.7% Chinook or Galena hops per compost dry matter (68,068 ppm) and then amended with 6 mL of water to achieve delivery of 8168 or 6848 ppm of α-acid and 2042 or 6126 ppm of β-acid, respectively. The mixture was then transferred to 50-mL plastic tubes (six tubes/treatment to achieve three tubes/treatment per each sampling on days 0 and 14 of incubation). Control litter not treated with either of the hops was prepared and similarly distributed to six tubes. Tubes were closed with screw caps and sealed with parafilm immediately upon receiving the litter preparations and then incubated at successive three-day temperature increments at 22 °C, 30 °C, and 37 °C and then for four days 42 °C, respectively, to simulate conditions commonly encountered during composting. For all incubations, the tubes were placed in BBL Anaerobic Gas Pack (Becton Dickinson) containers and incubated aerobically during days 0 to 3, then flushed with 100% with carbon dioxide and incubated under this atmosphere during days 3 to 14.

In Experiment 3, the screened poultry litter from the second collected batch was blended 3:1 with the ground bluestem hay, then 11 g of the poultry litter/bluestem hay mixture was mixed by hand without or with 0.7% of Chinook or Galena hops per compost dry matter and amended with 6 mL of water to achieve delivery of 8168 or 6848 ppm α-acid and 2042 or 6126 ppm β-acid, respectively. The resultant poultry litter/bluestem hay mixtures were transferred to 50-mL tubes, closed, and incubated, as described in Section 3.2.

### 2.3. Analytical

Upon collection of the triplicate sets of control and hop-treated tubes at their respective sampling times in Experiment 1, 20-mL of 0.4 M sodium phosphate buffer (pH 6.5) were added to each tube and vortexed to dislodge solids-associated microbes. For Experiments 2 and 3, collected compost tubes were each vortexed and transferred to separate 250-mL containers, each containing 90 mL of 0.4 M sodium phosphate buffer (pH 6.5) so as to accommodate the more absorptive capacity of the compost material in Experiments 2 and 3. After vortexing again, 4 mL of fluid were withdrawn from the collected tubes. One (1) mL of the collected fluid was used to prepare serial 10-fold dilutions for immediate bacteriological enumerations as described below. The remaining 3 mL of collected fluid was centrifuged at 10,000× *g* for 20 min. The resultant supernatants were stored at −20 °C until later colorimetric analysis of uric acid (Sigma-Aldrich, St. Louis, MO, USA), urea (BioAssay Systems, Hayward, CA, USA), and ammonium concentrations [36] and, in experiments 2 and 3, for gas chromatographic measurement of volatile fatty acid concentrations [37].

The packed centrifugation residue from Experiment 1 samples were analyzed for potential treatment effects on microbial community diversity using denaturing gradient gel electrophoresis, as described earlier [19]. The sequences of the bacteria-specific PCR primers used for amplification were 5′-ATTACCGCGGCTGCTGG-3′ and 5′-CGCCCGCCGCGCGCGGCGGGCGGGGCGGGGGCACGGGGGGCCTACGGGAGGCAGCAG-3′). For microbial enumeration of fluid samples collected at indicated time points in Experiment 1, these fluids were serially diluted out to 10^−^^6^ in 0.4 M sodium phosphate buffer (pH 6.5). The serial dilutions were plated onto Brilliant Green Agar (Oxoid Ltd., Basingstoke, Hampshire, UK) supplemented with 20 µg of novobiocin/mL and 25 µg of nalidixic acid/mL for enumeration of the novobiocin and nalidixic acid-resistant challenge strain of *S.* Typhimurium, as well as onto *E. coli*/Coliform and Aerobic petrifilm (3M Petrifilm, St. Paul, MN, USA) for enumeration of wildtype *E. coli* and total aerobes. For microbial enumeration of fluid samples collected at indicated time points in Experiments 2 and 3, serial ten-fold dilutions of fluid samples, prepared as described above, were spread to m-*Enterococcus* agar, BBL™ Mannitol Salt agar and Difco Rogosa SL agar (Becton, Dickinson) for selective enumeration of wildtype enterococci, staphylococci, and lactic acid bacteria, respectively. Serial dilutions were also plated onto *E. coli*/Coliform Count, Aerobic Count and Yeast and Mold Count Plates (3M Petrifilm, St. Paul, MN, USA) for enumeration of culturable coliform and aerobic bacteria, as well as culturable yeasts and molds. Inoculated plates and Petrifilm were incubated according to manufactures instructions. Ten-fold serial dilutions of control and hops-treated compost samples from Experiments 2 and 3, which were not inoculated with the novobiocin and nalidixic acid resistant challenge strain of *S.* Typhimurium, were also plated onto Brilliant Green Agar supplemented with 20 µg of novobiocin/mL for quantitative recovery of wildtype salmonellae. However, viable salmonellae were not recovered from any of these plated samples.

### 2.4. Statistics

Compost material was prepared and randomly distributed to each tube, as well as incubated and sampled independent of compost material within other tubes, and thus each tube served as an independent experimental unit. In all experiments, concentrations of uric acid, urea, and ammonia, expressed as µmol/g compost dry matter (DM), and log_10_ transformations of the microbial populations, were tested for main effects of hops treatment, day of compost, and their potential interaction using a two-way factorial design with the analysis of variance procedure of Statistix10 Analytical Software (Tallahassee, Florida, USA). When the main effects were significant at *p* < 0.05, a LSD All-Pairwise Comparisons Test was performed to test for differences between treatment means. For all comparisons, treatment differences were considered significant at *p* ≤ 0.05, and differences were considered tending for significance at 0.05 > *p* ≤ 0.10. Differences in bacterial population diversity in the DGGE profiles obtained from composts Experiment 1 were compared by analysis of band patterns. Diversity comparisons are presented descriptively by showing Dice percentage similarity coefficients (%SC) and dendrograms constructed by an unweighted pair group method using the arithmetic averages (UPGMA) options in Gel Compare II 6.6 (Applied Maths, Inc., Austin, TX, USA).

## 3. Results

### 3.1. Experiment 1

**Figure 1:**
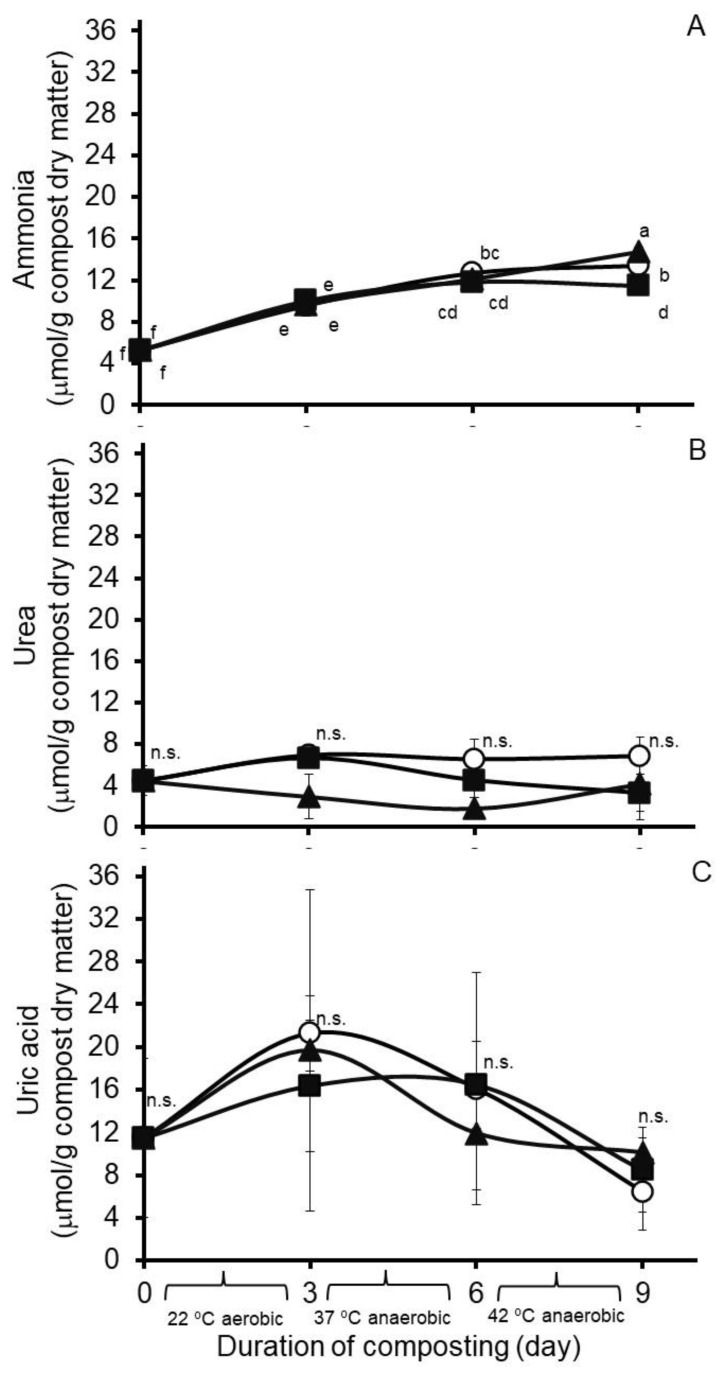
Accumulations in Experiment 1 of ammonia (**A**), urea (**B**) and uric acid (**C**) during ninedays of simulated composting of wood chip poultry litter at 22, 37 and 42 °C. Composts were treated without (circles) or with 0.26% (*wt*/*wt*) Chinook (squares) or 0.09% (*wt*/*wt*) Galena (triangles) hops preparations. Values are the least squares means from the treatment by day of compost interaction ± their standard deviations from *n* = 3 replicated composts at each sample time. Means with unlike lowercase letters differ (*p* < 0.05), based on a LSD all-pairwise comparisons test; means not differing significantly are denoted by n.s.

Results from an analysis of variance of Experiment 1 are presented in Table 1. Least squares means from the treatment by day of compost interactions reveal variable effects of hops treatment on nitrogen concentrations in the composted poultry litter (Figure 1). In the case of ammonia, a treatment by day of compost interaction was observed (standard error of the mean, SEM = 0.380). As illustrated in Figure 1A, least square mean ammonia concentrations increased each day during composting, and after nine days of composting, they were modestly lower in the compost treated with Chinook hops than in nontreated or Galena hops-treated composts. A main effect of treatment (SEM = 0.561) was observed on urea accumulations, but significant effects of day of compost (SEM = 0.601) or a treatment by day of compost interaction (SEM = 1.041) were not observed. The main effect treatment means for urea accumulations were highest for samples from the nontreated compost, lowest in samples from the Galena-treated compost, and intermediate in samples from the Chinook-treated compost (6.20, 4.71 and 3.34 µmol/g compost dry matter, respectively). A day of compost effect was observed on concentrations of uric acid (SEM = 2.429), but not as a main effect of treatment (SEM = 1.949) or a treatment by day of compost interaction (SEM = 4.207). The main effect days of compost means for uric acid concentrations in the compost were highest on day 3 (19.11 µmol/g DM), lowest on days 0 and 9 (11.49 and 8.35 µmol/g DM, respectively), and intermediate on day 6 (14.83 µmol/g DM). For comparison, least squares means of the treatment by day of compost interaction are presented for ammonia, urea, and uric acid concentrations in Figure 1.

**Figure 2:**
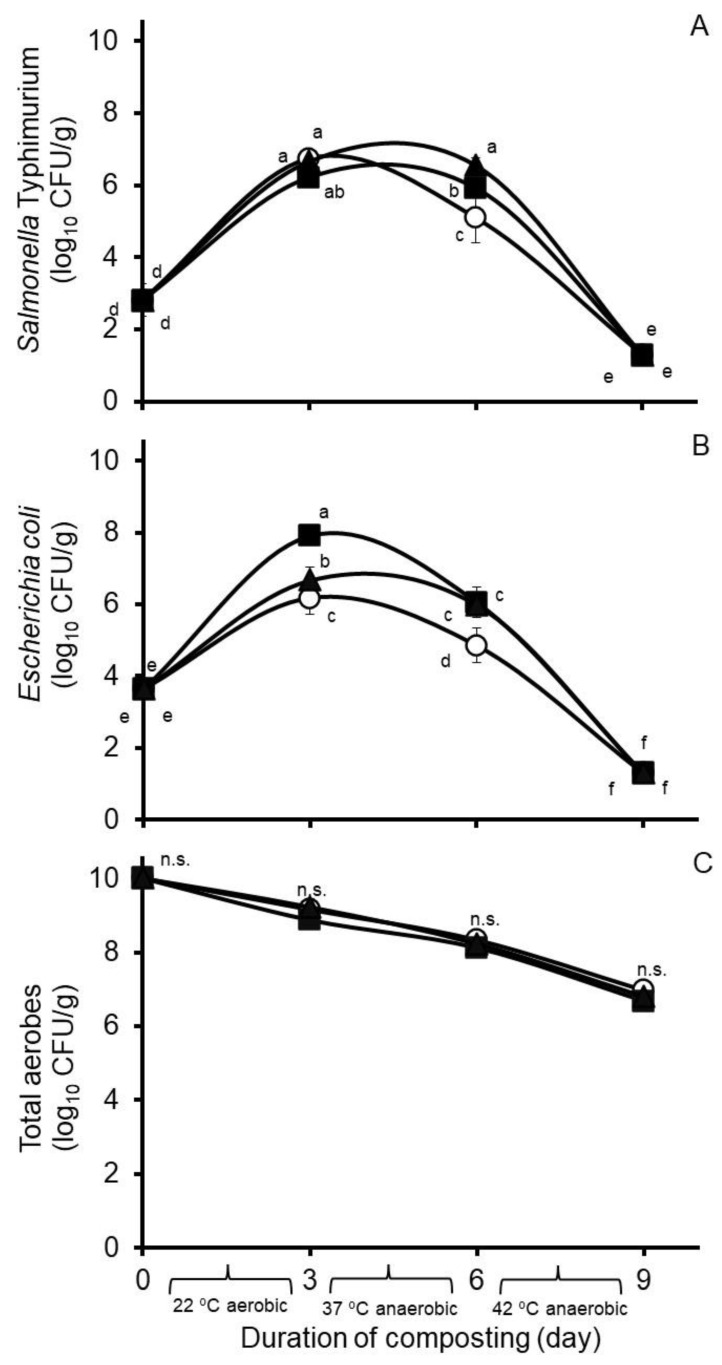
Concentrations in Experiment 1 of *Salmonella* Typhimurium (**A**), *Escherichia coli* (**B**), and total aerobes (**C**) during nine days of simulated composting of wood chip poultry litter at 22, 37, and 42 °C. Composts were treated without (circles) or with 0.26% (*wt*/*wt*) Chinook (squares) or 0.09% (*wt*/*wt*) Galena (triangles) hops preparations. Values are the least squares means from the treatment by day of compost interaction ± their standard deviations from *n* = 3 replicated composts at each sample time. Means with unlike lowercase letters differ (*p* < 0.05) based on a LSD all-pairwise comparisons test; means not differing significantly are denoted by n.s.

Effects of hops treatment on select bacteria populations in Experiment 1 are shown in Figure 2. Treatment by day of compost interactions were observed against concentrations of the challenge *S.* Typhimurium strain and wildtype *E. coli* (SEM = 0.199 and 0.147, respectively), but not against concentrations of wildtype aerobes (SEM = 0.117). A main effect of treatment was not observed against concentrations of total aerobes in the composts (SEM = 0.059); however, a day of compost effect was observed (SEM = 0.068), with concentrations of total aerobes decreasing in descending order during each day of composting (Figure 2C).

**Figure 3:**
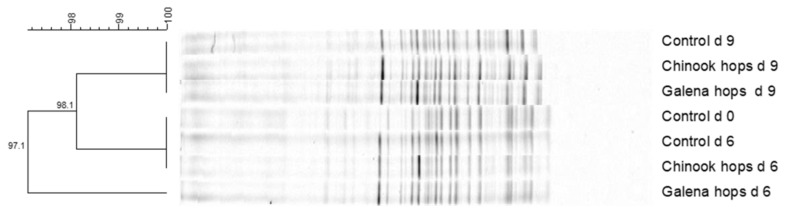
Denaturing gradient gel electrophoresis dendrogram from Experiment 1 showing effects of 0.26% (*wt*/*wt*) Chinook or 0.09% (*wt*/*wt*) Galena hops treatment on microbial diversity of composting poultry litter. Percentage similarity coefficient (the bar) between bands is considered likely the same or identical if ≥95, very similar if between 90 to 94, considered similar if between 85 to 89, and considered somewhat similar if between 80 to 84. It is not considered similar if ≤79.

Results from denaturing gradient gel electrophoresis diversity analysis reveal little if any appreciable effect of hops treatment or day of compost on microbial diversity in the hops extract-treated composts, as the similarity coefficients between bands exceeded 97% (Figure 3).

### 3.2. Experiment 2

**Figure 4:**
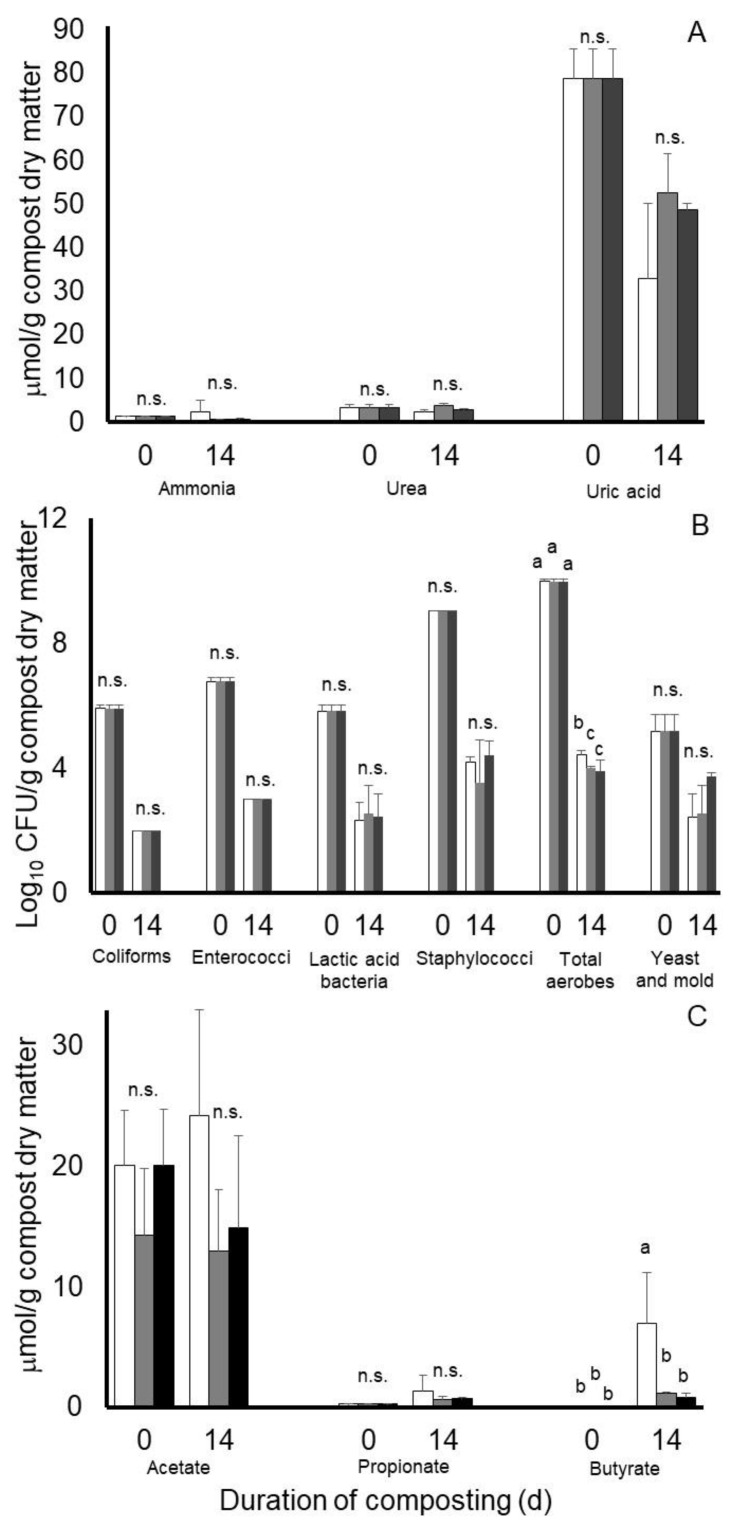
Least squares means of potential treatment by day of compost interactions for nitrogen (**A**), microbiological (**B**), and predominant volatile fatty acid accumulations (**C**) during 14 days of simulated composting of wood chip poultry litter treated without (open bars) or with 0.7% Chinook (gray bars) or Galena (black bars) hops in Experiment 2. Error bars reflect standard deviations from *n* = 3 replicated composts of each treatment and sample time. Means with unlike lowercase letters differ (*p* < 0.05) based on a LSD all-pairwise comparisons test; means not differing significantly are denoted by n.s.

Results from an analysis of variance of Experiment 2 are presented in Table 2. Least squares means of potential treatment by day of compost interactions for nitrogen, microbial, and volatile fatty acid characteristics of the composts of Experiment 2 are illustrated in Figure 4. Main effects of treatment (SEM = 0.462 and 0.242), day of compost (Table 2), or a treatment by day of compost interaction (SEM = 0.643 and 0.312) were not observed on concentrations of ammonia or urea, respectively, during the 14-day compost period of Experiment 2. Conversely, main effects of day of compost (Table 2 and Table 3), but not treatment (SEM = 4.013) or a treatment by day of compost interaction (SEM = 0.224), were observed on concentrations of uric acid in Experiment 2. Main effects of day of compost were observed on each microbial population measured in Experiment 2, with concentrations being decreased at the end of the 14-day compost period compared to initial concentrations (Table 3). Main effects of treatment on populations of coliforms, (SEM = 0.027), enterococci (SEM = 0.028), lactic acid bacteria (SEM = 0.211), staphylococci, (SEM = 0.237), total aerobes (SEM = 0.082), and yeast and mold (SEM = 0.273) were not observed. Likewise, treatment by day of compost interactions were not observed for coliforms (SEM = 0.041), enterococci (SEM = 0.043), lactic acid bacteria (SEM = 0.321), staphylococci (SEM = 0.338), and yeast and mold (SEM = 0.361). A treatment by day of compost interaction was observed, however, for populations of total culturable aerobes (SEM = 0.097) (Figure 4B). In this case, counts were decreased from pre-compost levels of 9.99 log_10_ CFU/g compost dry matter by nearly 6 log_10_ CFU units in the Galena- and Chinook-treated composts compared to a decrease of 5.56 log_10_ CFU/g observed in nontreated control composts.

Concentrations of volatile fatty acids in the poultry litter at the beginning of the compost period in Experiment 2 varied considerably with concentrations (means ± standard deviation) being 18.19 ± 5.17 and 0.28 ± 0.04 µmol/g compost dry matter for acetate and propionate, respectively. Concentrations of butyrate, isobutyrate, isovalerate, and valerate were near or below our detectable level of 0.10 µmol/g in the compost material at the start of composting. After 14 days of composting, a main effect of day of compost was observed on propionate concentrations (SEM = 0.182), with concentrations being 70% higher on day 14 than at the start of composting. Main effects of treatment (SEM = 0.222) or a treatment by day of treatment interaction (SEM = 0.315) were not observed on propionate concentrations. In the case of butyrate, a significant interaction (SEM = 0.902) was observed, with least square mean concentrations after 14 days of composting being remarkably higher in nontreated composts than in the Chinook or Galena hops-treated composts (7.02 versus 1.20 and 0.86 µmol/g compost dry matter, respectively) (Figure 4). Main effects of day of compost (SEM = 0.460) but not treatment (SEM = 0.871) were observed for butyrate. Main effects of treatment (SEM = 2.53, 0.076, 0.164 and 0.016), day of compost SEM = 2.089, 0.062, 0.132 and 0.013), or their interaction (SEM = 3.62, 0.107, 0.229 and 0.023) were not observed for acetate, isobutyrate, isovalerate, or valerate, respectively. Overall means (± standard deviations) for acetate, isobutyrate, isovalerate, and valerate were 17.35 ± 8.25, 0.14 ± 0.26, 0.41 ± 0.57, and 0.10 ± 0.04 µmol/g compost dry matter, respectively.

### 3.3. Experiment 3

**Figure 5:**
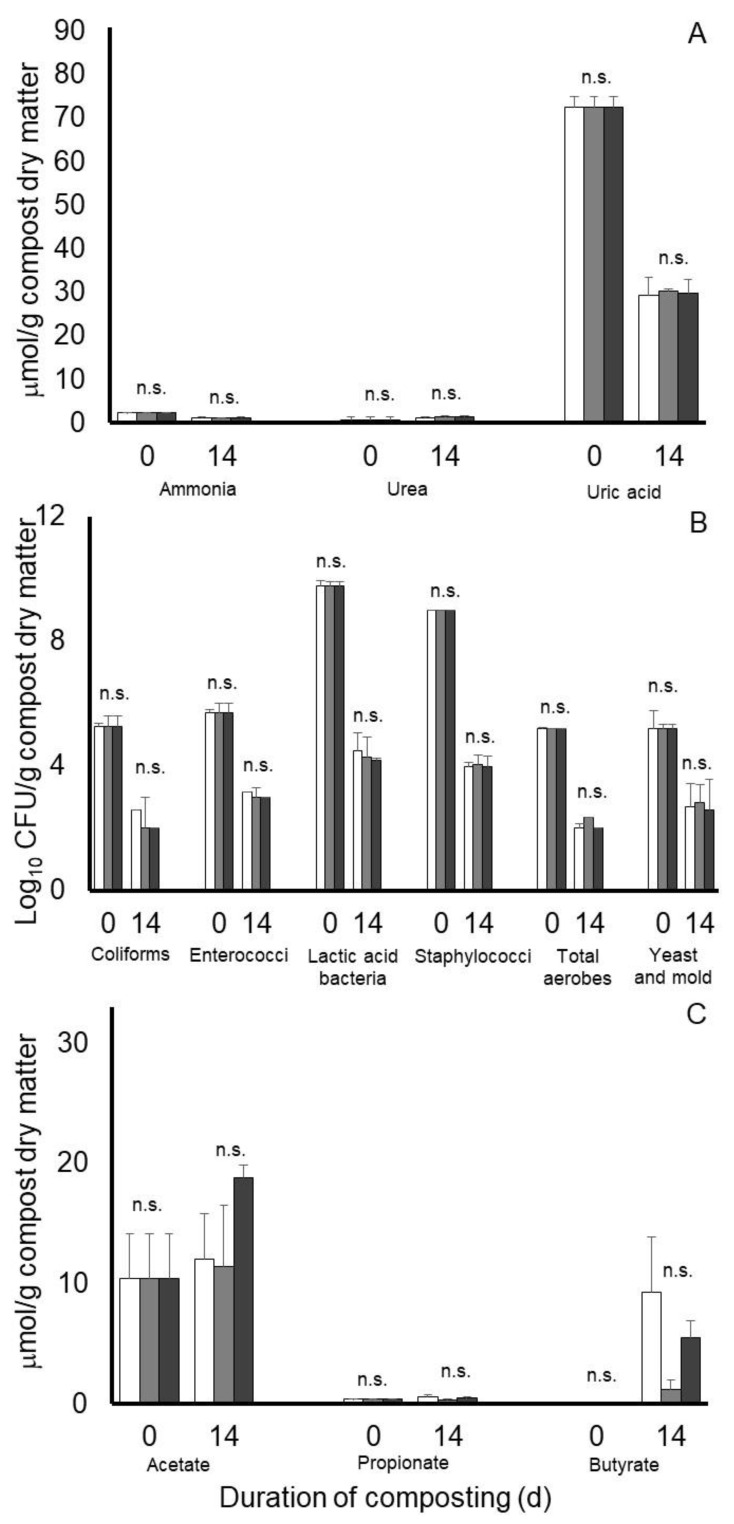
Least squares means of potential treatment by day of compost interactions for nitrogen (**A**), microbiological (**B**), and predominant volatile fatty acid accumulations (**C**) during 14 days of simulated composting of wood chip poultry litter mixed 3:1 with Bluestem hay and treated without (open bars) or with 0.7% Chinook (gray bars) or Galena (black bars) hops in Experiment 3. Error bars reflect standard deviations from *n* = 3 replicated composts of each treatment and sample time; means not differing significantly are denoted by n.s.

Results from an analysis of variance of Experiment 3 are presented in Table 2. A main effect of day of compost was observed on all nitrogen and microbiological measurements (Table 2 and Table 4), with concentrations on ammonia and uric acid being decreased, concentrations of urea being increased, and all microbial populations being decreased after 14 days of composting of the 3:1 poultry litter:bluestem hay mixture. Main effects of treatment (SEM = 0.058, 0 160 and 1.165) or treatment by day of compost interactions (SEM = 0.096, 0.273 and 2.014) were not observed on ammonia, urea, or uric acid accumulations, respectively, in the composts of Experiment 3. Similarly, main effects of treatment (SEM = 0.218, 0.091, 0.118, 0.089, 0.132 and 0.265) or treatment by day of compost interactions (SEM = 0.360, 0.154, 0.192, 0.153, 0.223 and 0.458) were observed for concentrations of coliforms, enterococci, lactic acid bacteria, staphylococci, total aerobes, and yeast and molds, respectively. Least squares means from the treatment by day of compost interactions for nitrogen and microbiological measurements of Experiment 3 are presented in Figure 5.

Concentrations of volatile fatty acids in the 3:1 poultry litter: bluestem hay mixture at the beginning of the compost period in compost Experiment 3 also varied considerably, with concentrations (means ± standard deviation) being 10.41 ± 2.87 and 0.42 ± 0.01 µmol/g compost dry matter for acetate and propionate, respectively. In this case, concentrations of butyrate, isobutyrate, isovalerate, and valerate in the compost material before composting were near or below our detectable concentration. After 14 days of composting, main effects of day of composting were observed for butyrate and isovalerate (SEM = 0.925 and 0.020, respectively), with concentrations (5.38 and 0.23 µmol compost dry matter, respectively) being higher on day 14 than at the start of composting. Main effects of treatment SEM = 0.010 and 0.026) or a treatment by day of compost interaction (SEM = 0.017 and 0.035) were not observed on valerate or isovalerate, respectively. Although, concentrations were <0.2 µmol/mL. There tended to be a main effect of treatment (*p* = 0.056, SEM = 1.257), as well as a treatment by day of composting interaction (SEM = 1.602) on butyrate concentrations (Figure 5C). In this case, butyrate concentrations after 14 days of composting tended to be higher in nontreated and Galena-treated composts than in Chinook-treated composts (9.34 and 5.57 versus 1.24 µmol/g compost dry matter, respectively). Main effects of treatment (SEM = 1.745, 0.044 and 0.664), day of compost (SEM = 1.521, 0.038 and 0.626) or their interaction (SEM = 2.634, 0.065 and 1.083) were not observed for acetate, propionate, and isobutyrate, respectively. After 14 days composting, respective concentrations (means ± standard deviation) of these acids were 14.06 ± 4.80, 0.49 ± 0.14, and 0.70 ± 1.87 µmol/g compost dry matter.

## 4. Discussion

Biotechnological processes, such as composting and ensiling, are recognized as economically effective way of decreasing concentrations of pathogenic and other unwanted microbes in poultry litter intended for use as a nitrogen fertilizer or as a crude protein feed supplement for ruminants [5,7,8,9,10,11]. Results from the present experiments are consistent with this understanding, as evidenced by the dramatic log-fold decreases in *S.* Typhimurium, *E. coli*, and certain Gram-positive bacteria, as well as yeast and mold populations achieved during the composting process. However, results from the present study failed to reveal appreciable effects of hops treatment on antimicrobial activity or prevention of uric acid or urea degradation during composting with administration of 79 ppm hops β-acid in the first study and 2042 and 6126 ppm hops β-acid provided, respectively, with the Chinook and Galena hops in the second and third studies. The lack of enhanced antimicrobial activity against the Gram-negative bacteria is not unexpected, as the hops β-acids, as reviewed by Fahle et al. [38], are recognized as having little, if any, antimicrobial activity against these bacteria. Conversely, hops’ β-acids and their derivatives have been demonstrated to exert antimicrobial activity against a variety of Gram-positive bacteria, such as species belonging to *Bacillus*, *Clostridium*, *Enterococcus*, *Listeria*, *Propionibacterium*, *Staphylococcus*, and *Streptococcus* [38]. Thus, the lack of enhanced antimicrobial activity against the Gram-positive bacteria measured in the present study was unexpected, but supported, by the lack of appreciable effect of hops treatment or day of composting on microbial diversity, as apparent in the denaturing gradient gel electrophoresis profile. For comparison, Flythe and colleagues reported that doses of 30 to 60 ppm of hops β-acids achieved inhibition of Gram-positive hyper ammonia-producing bacteria, as well as lactic acid-producing *Streptococcus bovis* during pure culture or in vitro incubation of rumen contents [23,34]. While the pH of the litter was not measured in the present studies, it is likely the pH of the composting litter may have been more alkaline and thus contributed, at least partially, to the lack of activity as the antimicrobial activity of hops β-acid is recognized as being more potent under acidic rather than alkaline conditions [38]. Poultry litter of this type has been reported to have a pH range between 6.8 to 7.8 [39] and exceeded pH 8.0 during three weeks of composting in the study reported by Kim et al. [40]. Flythe [23] also demonstrated that the hops β-acids-induced inhibition of the hyper ammonia-producing bacteria was greater at pH 5.6 than pH 6.7.

Despite the lack of an apparent antimicrobial-enhancing effect of the Chinook and Galena hops treatments on the microbial populations examined in the present composting experiments, we did observe treatment or tendencies for treatment effects on accumulations of certain volatile fatty acids during the composting process. For example, while total concentrations of volatile fatty acids, representing the sum of acetate, propionate, butyrate, isobutyrate, valerate and isovalerate, were respectively low at the beginning of the composting process in Experiments 2 and 3 (18.62 ± 5.16 and 10.89 ± 2.86 µmol/g compost material), concentrations of butyrate increased dramatically during composting in the untreated, but not in the hops-treated, compost mixtures. Thus, under the conditions of this study, the accumulation of butyrate in the untreated control composts over the 14-day compost period suggests the sustained presence of active butyrate-producing bacteria. This observation is not unexpected, as diverse populations of butyrate-producing bacteria have been reported in the avian gut and broiler litter [41,42]. When compared to the untreated control composts, butyrate accumulation at the end of the 14-day compost period decreased more than 5.8-fold in both the Chinook- and Galena-treated composts in Experiment 2 and by 7.5- and 1.7-fold, respectively, in Experiment 3. It is attractive to hypothesize that the decrease in butyrate production observed in the present studies was manifested by a decrease in certain butyrate-producing bacterial populations similar to that reported by Blatchford et al. [43], who observed decreased populations of butyrate-producing *Coprococcus* and *Eubacterium* in human fecal incubations treated with the equivalent of 100 ppm of hops extract or greater. Certain clostridial populations within Clusters IV and XIVa are also known to express butyrate-producing activity; however, Tillman et al. [25] reported no significant effect on these clostridial populations after providing chicks 22 days of unrestricted access to drinking water containing 125 ppm of the hops component lupolone (β-acids).

The hops effects could possibly reflect adverse effects on digestion of diet dry matter and crude protein, as reported by Lavrenčič and colleagues [44] during incubation of rumen contents supplemented with approximately 250 to 1125 ppm of freshly milled Aurora or Dana hops varieties. As alluded to earlier, the decrease in ruminal ammonia production has been demonstrated to be due to hops-induced inhibition of hyper ammonia-producing bacteria [23], although the effects on butyrate production are less clear. For instance, Flythe and Aiken [34] reported no significant inhibitory effect of a 30 ppm propylene glycol-β-hops extract treatment during mixed culture of rumen microbes or during pure culture of the lactate-utilizing ruminal bacterium *Megasphaera elsdenii* strain B159. Under certain conditions, *M. elsdenii* can be a major butyrate producer [45]. Conversely, the 30 ppm-hops treatment did inhibit growth of a major lactic acid producing rumen bacterium, *Streptococcus bovis* strain JB1, when similarly grown in pure culture [34]. Thus, it is possible that, when grown in mixed cultures, hops-induced inhibition of lactate-producing bacteria could indirectly decrease butyrate production by depriving lactate-utilizing bacteria, such as *M. elsdenii* of substrate used for growth and butyrate production. Narvaez et al. [46] reported reduced butyrate accumulations in cultures of mixed populations of rumen microbes with the addition of dried, unprocessed preparations of Cascade or Millennium, but not Teamaker hops varieties (each at 800 ppm). Thus, it is possible that the differential effects of the different hops preparations on butyrate production reflect a dose effect of the bioactive hops components, which may vary in concentration within the different preparations. Undoubtedly, further research is needed to more clearly define the role of hops treatments on butyrate-producing anaerobes as well as other important microbes present in composted poultry litter.

## 5. Conclusions

Results from the present study revealed little if any benefit of either Chinook or Galena hops treatment on the antimicrobial effectiveness or nitrogen retention in composting poultry litter. The hops treatments did, however, prevent the accumulation of butyrate in the chicken litter composted with or without bluestem hay, with both Chinook and Galena hops being near equally effective in this regard. From a practical perspective, the hops-induced inhibition of butyrate production in fermented feedstuffs, such as composted poultry litter or silage, could be beneficial in preventing adverse effects of butyrate on the palatability of these feedstuffs. An additional consideration, however, is that residual concentrations of hops within the lumen of the gut would not be high enough to adversely affect gastrointestinal butyric acid production, which is thought to be beneficial for gut health.

## Figures and Tables

**Table 1:** Analysis of variance results for effects of hops treatments, day of compost and their interactions on nitrogen and select microbiological populations after nine days of simulated composting of wood chip poultry litter mixed (Experiment 1) ^1^.

	F Statistic (*p*-Value)		
	Treatment	Day	Treatment by Day
Nitrogen measurements			
Ammonia	2.50 (0.0987)	261.6 (<0.0001)	5.22 (0.0015)
Urea	6.52 (0.0044)	0.80 (0.5039)	1.81 (0.1394)
Uric acid	0.03 (0.9710)	3.60 (0.0280)	0.29 (0.9341)
Microbiological measurements			
*Salmonella*	2.13 (0.1367)	467.7 (<0.0001)	4.11 (0.0056)
*Escherichia coli*	8.40 (0.0013)	840.8 (<0.0001)	11.11 (<0.0001)
Total aerobes	3.52 (0.0422)	402.3 (<0.0001)	0.63 (0.7018)
Degrees of Freedom	2	3	6

^1^ Concentrations of uric acid, urea, ammonia, expressed as µmol/g compost dry matter (DM), and log_10_ transformations of the microbial populations were tested for main effects of hops treatment, day of compost and their potential interaction using a two-way factorial design with the analysis of variance procedure of Statistix10 Analytical Software (Tallahassee, FL, USA).

**Table 2:** Analysis of variance results for effects of hops treatments, day of compost, and their interactions on nitrogen and select microbiological populations after 14 days of simulated composting of a 3:1 mixture of wood chip poultry litter mixed without (Experiment 2) or with ground bluestem hay (Experiment 3) ^1^.

	Experiment 2, F Statistic (*p*-Value)			Experiment 3, F Statistic (*p*-Value)		
	Treatment	Day	Treatment by Day	Treatment	Day	Treatment by Day
Nitrogen measurements						
Ammonia	1.19 (0.3324)	0.00 (9570)	1.23 (0.3260)	0.90 (0.4351)	230.67 (<0.0001)	0.55 (0.5952)
Urea	1.84 (0.1945)	1.83 (0.2007)	2.15 (0.1595)	0.27 (0.7695)	10.46 (0.0102)	0.15 (0.8617)
Uric acid	1.67 (0.2230)	60.98 (<0.0001)	1.89 (0.1941)	0.07 (0.9354)	809.25 (<0.0001)	0.04 (0.9634)
Microbiological measurements						
Coliforms	0.00 (1.000)	13,483.23 (<0.0001)	0.00 (1.000)	0.83 (0.4628)	130.64 (<0.0001)	0.50 (0.6217)
Enterococci	0.00 (1.000)	11,649.84 (<0.0001)	0.00 (1.000)	0.38 (0.6952)	531.49 (<0.0001)	0.21 (0.8107)
Lactic acid bacteria	0.06 (0.9452)	168.58 (<0.0001)	0.05 (0.9525)	0.97 (0.4021)	456.55 (<0.0001)	0.60 (0.5694)
Staphylococci	0.93 (0.4183)	327.88 (<0.0001)	0.92 (0.4259)	0.12 (0.8912)	1924.19 (<0.0001)	0.06 (0.9380)
Total aerobes	2.86 (0.0906)	55,32.72 (<0.0001)	4.15 (0.0425)	0.50 (0.6178)	1080.86 (<0.0001)	0.29 (0.7534)
Yeast and mold	1.77 (0.2058)	59.74 (<0.0001)	2.04 (0.1733)	0.09 (0.9158)	54.49 (<0.0001)	0.05 (0.9525)
Volatile fatty acid accumulations						
Acetate	2.87 (0.0900)	0.08 (0.7808)	0.85 (0.4521)	2.02 (0.1795)	3.46 (0.0960)	1.46 (0.2833)
Propionate	0.70 (0.5128)	6.25 (0.0279)	0.67 (0.5311)	2.26 (0.1501)	2.32 (0.1620)	1.70 (0.2361)
Butyrate	3.08 (0.0801)	23.25 (0.0005)	9.45 (0.0041)	3.80 (0.0556)	20.16 (0.0015)	3.84 (0.0621)
Isobutyrate	0.99 (0.3957)	2.76 (0.1228)	0.99 (0.4000)	0.95 (0.4156)	0.75 (0.4102)	0.59 (0.5759)
Isovalerate	1.13 (0.3502)	2.40 (0.1476)	1.16 (0.3470)	3.38 (0.0716)	49.17 (0.0001)	3.13 (0.0930)
Valerate	0.24 (0.7917)	0.00 (0.9638)	0.21 (0.8128)	0.91 (0.4306)	19.65 (0.0016)	0.56 (0.5908)
Degrees of freedom	2	1	2	2	1	2

^1^ Concentrations of uric acid, urea, ammonia, volatile fatty acids, expressed as µmol/g compost dry matter (DM) and log_10_ transformations of the microbial populations were tested for main effects of hops treatment, day of compost, and their potential interaction using a two-way factorial design with the analysis of variance procedure of Statistix10 Analytical Software (Tallahassee, Florida, USA).

**Table 3:** Main effect means of day of composting in Experiment 2 on concentrations of nitrogen and select microbiological populations after 14 days of simulated composting of wood chip poultry litter ^1^.

	Nitrogen Characteristics (µmol/g Compost Dry Matter)			Microbiological Characteristics (log_10_ Colony Forming Units/g Compost Dry Matter)					
	Ammonia	Urea	Uric Acid	Coliforms	Enterococci	Lactic Acid Bacteria	Staphylococci	Total Aerobes	Yeast and Mold
Pre-compost	1.32 ^n.s.^	3.40 ^n.s.^	78.89 ^a^	5.92 ^a^	6.79 ^a^	5.83 ^a^	9.05 ^a^	9.99 ^a^	5.18 ^a^
Ending (day 14)	1.29	3.05	44.80 ^b^	2.00 ^b^	3.00 ^b^	2.43 ^a^	4.06 ^a^	4.11 ^a^	2.90 ^b^
SEM ^2^	0.371	0.183	3.087	0.024	0.025	0.185	0.195	0.056	0.208

^1^ Composts were incubated at successive three-day increments at 22 °C, 30 °C, and 37 °C and then for four days at 42 °C, respectively, to simulate conditions commonly encountered during composting. ^2^ SEM; Standard error of the mean. ^a,b^ Means within columns with unlike superscripts differ at *p* < 0.05 based on a LSD all pair-wise comparisons test; means not differing significantly are denoted by n.s.

**Table 4:** Main effect means of day of composting on concentrations in Experiment 3 of nitrogen and select microbiological populations after 14 days of simulated composting of a 3:1 mixture of wood chip poultry litter and ground bluestem hay ^1^.

	Nitrogen Characteristics (µmol/g Compost Dry Matter)			Microbiological Characteristics (Log_10_ Colony Forming Units/g Compost Dry Matter)					
	Ammonia	Urea	Uric Acid	Coliforms	Enterococci	Lactic Acid Bacteria	Staphylococci	Total Aerobes	Yeast and Mold
Pre-compost	2.29 ^a^	0.74 ^b^	72.50 ^a^	5.25 ^a^	5.69 ^a^	5.18 ^a^	9.00 ^a^	9.77 ^a^	5.21 ^a^
Ending (day 14)	1.21 ^b^	1.40 ^a^	29.80 ^b^	2.19 ^b^	3.05 ^b^	2.11 ^b^	3.99 ^b^	4.31 ^b^	2.69 ^b^
SEM ^2^	0.055	0.158	1.163	0.208	0.089	0.111	0.089	0.129	0.265

^1^ Composts were incubated at successive three-day increments at 22 °C, 30 °C, and 37 °C and then for four days at 42 °C, respectively, to simulate conditions commonly encountered during composting. ^2^ SEM; Standard error of the mean. ^a,b^ Means within columns with unlike superscripts differ at *p* < 0.05 based on a LSD all pair-wise comparisons test.

## Data Availability

The data presented in this study are available upon request from the corresponding author.
